# Notes and News

**Published:** 1895-06-22

**Authors:** 


### Notes and News.

(Collected and Communicated.)

Professor Leyden has received the Order of St. Anne of the first class from Czar Nicholas, in recognition of his services in attendance upon the late Czar Alexander.

In connexion with the festival dinner of the Westminster Hospital to be held on July 6th, H.R.H. the Duke of Connaught in the chair, upward of £2,100 has already been received.

A new mountain health resort has been opened up to consumptives in America. It is situated in the Northern Adirondack region, where a sanatorium with one hundred acres of land attached is to be opened under the charge of Sisters of Mercy. A large central hospital with outlying cottages has been planned. A sun bath portico will be provided. Close to the institution are thousands of acres of pine forest.

Public attention has been so centred on the subject of our hospitals during the past week, owing to the event of the Hospital Sunday Fund collection, that it is interesting to be able to bring out an example showing the goodwill and gratitude which exist in the minds of the people towards hospitals, although the proof of this is not always so tangibly shown, as in the present instance. The treasurer of the Westminster Hospital has just received the following letter: "Herewith please find cheque value £10, being a thank-offering to God for preservation of my late husband's life, William Pearce, who met with a railway accident at the Old Kent Road Station on March 24th, 1870, and was conveyed to your hospital, where he received the most kind and skilful attention, with careful and best of medical treatment. By the blessing of God he was enabled to leave your hospital at the end of three weeks. My husband fell on sleep nine years ago. I have long purposed in my heart, should I have it ever in my power, to send a donation, which with much pleasure am doing. Commending your hospital and similar institutions to our heavenly Father, I remain, dear sir," &c.

Mrs. Caroline M. Jennes has bequeathed 25,000 dols. for an endowment fund of the Hahnemann Hospital, Philadelphia.

Mr. A. Liste Webb, M.R.C.S., M.R.C.P., has been appointed resident medical officer to the National Hospital for Diseases of the Heart and Paralysis *vice* Mr. J. W. Goldney, whose time has expired.

With a view to making a strong effort to raise the Liberator Relief Fund to £100,000, it is proposed to have a Liberator Sunday on the first Sunday in July. All ministers and congregations are invited to co-operate.

The Melbourne Hospital is in debt to the amount of £14,000, and at a meeting held in the Town Hall on April 24th (the Mayor in the chair), it was resolved to have a "week of self-denial" for the purpose of collecting funds.

A measure has been placed before the Legislature of the State of New York which provides for the provision and treatment of poor persons bitten by rabid animals. There is a Pasteur Institute in New York, to which such patients would be conveyed.

A convalescent home for the police of Scotland has just been opened by the Countess of Moray, at St. Margaret's Bay, near Inverkeithing. The home is prettily situated, and sheltered by the Ferry hills, and the Castland woods. The accommodation provides for six inmates.

Crushed wood fibre has been adapted to the making of splints by Dr. Tracey, of Boston, U.S.A. The material is extremely light, strong, clean, and adaptable. The new appliance was shown at the recently-held annual meeting of the American Medical Association at Baltimore.

We regret to announce the death of Sir George Porter, who may be regarded as the leading surgeon of Ireland. He was Regius Professor of Surgery, University of Dublin, senior surgeon to the Meath Hospital, and consulting surgeon to many other hospitals, and held the post of Surgeon-in-Ordinary to the Queen in Ireland since 1869.

In answer to an inquiry regarding the duty of revising the certificates of medical men sought to be imposed on their medical officer, Dr. Waldo, by the Vestry of St. George, Southwark, the Local Government Board say:—On the general question the Board would observe that as regards duties of a somewhat analogous character, which at times devolve on the Medical Officer of Health under the Infectious Disease (Notification) Act, they have laid it down that even when question arises as to the good faith of a certifying medical practitioner, the Medical Officer of Health should, if it becomes necessary to revise a certificate, not only seek the co-operation of the medical practitioner concerned, but should also observe, as far as possible, the customs that usually govern the relations of medical practitioners to each other. On the particular case submitted in your letter of the 23rd ult., the Board do not gather that the undertaking entered into by Dr. Waldo, "to attend, if required," on certain employés "during illness," involves any obligation on his part to revise either the diagnosis or the certificates granted by other medical practitioners who have been called in to attend on such persons.

